# Screening of Supports for the Immobilization of **β**-Glucosidase

**DOI:** 10.4061/2011/642460

**Published:** 2011-09-11

**Authors:** Joelise de Alencar Figueira, Fernanda Furlan Gonçalves Dias, Hélia Harumi Sato, Pedro Fernandes

**Affiliations:** ^1^Department of Bioengineering, Higher Technical Institute (IST), Avenida Rovisco Pais, 1049-001 Lisboa, Portugal; ^2^Centre for Biological and Chemical Engineering, Institute for Biotechnology and Bioengineering (IBB), IST, Avenida Rovisco Pais, 1049-001 Lisboa, Portugal; ^3^Laboratory of Food Biochemistry, Department of Food Science, School of Food Engineering, University of Campinas-UNICAMP, Avenida Monteiro Lobato 80, C. P. 6121, 13083-862, Campinas, SP, Brazil

## Abstract

A set of supports were screened for the immobilization of a partially purified extract of *β*-glucosidase from *Aspergillus* sp. These supports, namely, Eupergit, Amberlite, alginate, gelatin, polyvinyl alcohol- (PVA-) based matrices (Lentikats), and sol-gel, have proved effective for the implementation of some other enzyme-based processes. The initial criterion for selection of promising supports prior to further characterization relied on the retention of the catalytic activity following immobilization. Based on such criterion, where immobilization in sol-gel and in Lentikats outmatched the remaining approaches, those two systems were further characterized. Immobilization did not alter the pH/activity profile, whereas the temperature/activity profile was improved when sol-gel support was assayed. Both thermal and pH stability were improved as a result of immobilization. An increase in the apparent *K*_*M*_ (Michaelis constant) was observed following immobilization, suggesting diffusion limitations.

## 1. Introduction

*β*-Glucosidases (*β*-D-glucoside glucohydrolases, EC 3.2.1.21) are enzymes that transfer a glycosyl group between oxygen nucleophiles. They are, therefore, accountable for the hydrolysis of *β*-glycosidic linkages in amino-, alkyl-, or aryl-*β*-D-glucosides, cyanogenic glycosides, and di- and short chain oligo-saccharides [1, 2]. *β*-glucosidases can be used in the production of aromatic compounds, in the stabilization of juices and beverages, and in the improvement of the organoleptic properties of food and feed products; they are also used in biomass degradation, in the production of fuel ethanol from cellulosic agricultural residues, and in the synthesis of alkyl- and arylglycosides from natural polysaccharides or their derivatives and alcohols, by reversed hydrolysis or trans-glycosylation, leading to products with applications in pharmaceutical, cosmetic, and detergent industries [1, 3–5]. The immobilization of *β*-glucosidase in a solid carrier offers the prospect of cost savings and widens the flexibility of process design, by enabling continuous operation (or multiple cycles of batch operation on a drain-and-fill basis) and simplifying downstream processing. Enzyme immobilization also allows for a high-biocatalyst load within the bioreactor, thus leading to high-volumetric productivities [6, 7]. Guidelines for cost analysis of bioconversion processes have been recently suggested [8]. In the present work, several immobilization methods were screened as suitable approaches for the immobilization of a *β*-glucosidase from an *Aspergillus* sp. Specifically, immobilization in calcium alginate, in Eupergit, in gelatin, in glutaraldehyde-activated Amberlite, in polyvinyl alcohol-(PVA-) based matrices (Lentikats), and in sol-gel was evaluated, since these approaches have been shown to provide convincing approaches for the design of different bioconversion systems anchored in immobilized biocatalysts [6–10]. The primary screening criterion relied on the determination of the relative activity after immobilizations. According to such feature, the most promising results were obtained when *β*-glucosidase was immobilized in either lens-shaped Lentikats or in a tetramethoxysilane- (TMOS-) based xerogel support. These were,therefore, selected for more detailed studies. Within the authors' knowledge, immobilization of *β*-glucosidase in Lentikats supports has not been reported yet, and references to immobilization in TMOS-based supports are relatively scarce [11–13]. Lentikats technology is relatively recent [14] but has been proving effective for the immobilization of enzymes targeted for applications in food and feed and pharmaceutical industries, such as oxynitrilase [15], penicillin acylase [16], dextransucrase [17], glucoamylase [18], invertase [19], and galactosidase [20]. The application of sol-gel methodologies for the immobilization of enzymes is also relatively recent [21] but has expanded rapidly [22]. Accordingly, several enzymes have been immobilized using this method [23], among them lipase [24, 25], penicillin acylase [26], and horseradish peroxidase [27].

In the present work, when the effect of the pH in biocatalyst activity was assessed, no influence resulting of immobilization was evident. On the other hand, when the effect of temperature in enzyme activity was assessed, sol-gel immobilization did not lead to a change in the optimal temperature but apparently minimized thermal deactivation for temperatures in excess of 60°C. This pattern was also observed when the thermal stability was assessed. Lentikats could not be used for temperatures in excess of 55°C, due to melting of the support, a feature previously reported [18, 19]. An increase in the apparent *K_M_* (Michaelis constant) was observed following immobilization, suggesting diffusion limitations. Both methods allowed for consecutive 15 minutes batch runs without decay in catalytic activity.

## 2. Materials and Methods

### 2.1. Chemicals

LentiKat liquid, a PVA-based material, and LentiKat stabilizer came from GeniaLab (Braunschweig, Germany), tetramethoxysilane (TMOS) ≥99%, 4-Nitrophenyl-*β*-D-glucopyranoside (p-NPG), sodium alginate, and Amberlite IRC86 were from Sigma Aldrich (St. Louis, USA), and Amberlite IRC 50 was from Rohm and Haas (Darmstadt, Germany). Eupergit C and Eupergit C 250 L were a kind gift of Evonik Röhm GmbH (Darmstadt, Germany). All solutions were prepared in distilled water. All other chemicals used were of analytical grade from various suppliers.

### 2.2. Production of *β*-Glucosidase

A fungal *Aspergillus sp. *strain belonging to the culture collection of the Biochemistry and Food Laboratory, Faculty of Food Engineering, State University of Campinas, Brazil, was used as source of *β*-glucosidase. The fungi were grown in potato dextrose agar slant tubes and kept in a protective layer of Vaseline during storage. Spores were then spread on Petri dishes containing agar potato dextrose and incubated for 5 days at 30°C.

The culture medium used for the production of the enzyme was prepared from a mixture of (g) wheat bran (95) and sugar cane bagasse (5) in 100 mL distilled water. After thorough mixing, amounts of 20 g of culture medium were transferred into 500 mL Erlenmeyer flasks and the whole sterilized in an autoclave (20 minutes, 121°C). 10 mm discs were taken from the Petri dish cultures, and 15 disks were transferred to each Erlenmeyer flask containing the culture medium. The Erlenmeyer flasks were incubated for 5 days at 30°C.

Enzyme extraction from the cultures was performed by adding 100 mL of distilled water to the Erlenmeyer flasks and shaking at 150 rpm for 20 minutes. The resulting suspension was filtered through filter paper. Salting out from the filtrate was carried out by adding an ammonium sulfate solution (80% of saturation) and storing at 3°C overnight. The suspension was centrifuged for 10 minutes at 10000 rpm, and the precipitate was suspended in sodium phosphate buffer 0.05 M pH 7.0. This extract was lyophilized for 48 hours and was stored in a refrigerator at 4°C.

### 2.3. Screening of Supports for *β*-Glucosidase Immobilization

#### 2.3.1. *β*-Glucosidase Immobilization in Polyvinyl Alcohol—Lentikats

The enzyme preparation was diluted 1000-fold in 100 mM acetate buffer pH 4.5. Immobilization in Lentikats was performed according to the protocol provided by GeniaLab (http://www.genialab.de/download/tt-english.pdf, assessed on the 21st March, 2011), adding 0.1 mL of the diluted enzyme preparation to 1 mL of LentiKat liquid. The resulting solution was extruded to Petri dishes. After dehydration, under 30°C, to 30% (w/w) of the original weight to allow for gelation, the resulting lens-like particles were incubated in 100 mL of a 15 GL^−1^ solution of LentiKat Stabilizer for two hours at room temperature. The lenses were then washed and stored in 100 mM acetate buffer pH 4.5 at 4°C until use.

#### 2.3.2. *β*-Glucosidase Immobilization in Sol-Gel

Immobilization in sol-gel was performed as described elsewhere [28]. Briefly, 0.16 mL of the diluted enzyme preparation were mixed with a solution containing 100 *μ*L TMOS and 40 *μ*L HCl (10 mM), which had been previously sonicated in a Transsonic T 460 sonicating water bath for 10 min. The sol-gel solution thus obtained was immediately added to 6 mL of a 150 mM AOT/isooctane solution. The mixture was vortexed for 1 min, washed twice with 100 mM acetate buffer pH 4.5, and aged under room temperature and controlled water activity, *a*_w_ = 0.75, for one week. The particles obtained, with size under 100 *μ*m [28], were suspended in 1 mL of the same acetate buffer and stored at 4°C until use.

#### 2.3.3. *β*-Glucosidase Immobilization in Calcium Alginate

*β*-Glucosidase was immobilized in calcium alginate as described by Kawaguti et al. [29, 30] with modifications. Briefly, the enzyme was added to a sterile solution of sodium alginate (3%). After thorough mixing, the resulting solution was extruded to a sterile calcium chloride solution (0.3 M). The resulting beads were recovered by filtration, transferred to the calcium chloride solution, and hardened by incubating at 4°C for about 2 hours. The beads were thoroughly washed with distilled water for the removal of excess calcium chloride and used for the determination of activity.

#### 2.3.4. *β*-Glucosidase Immobilization in Eupergit C and Eupergit 250 L

200 mg of Eupergit were washed with 5 mL of distillated water. The suspension was centrifuged (10 minutes, 4000 rpm), and the supernatant was disposed of 5 mL of enzyme solution (0.1 g/L) in pH 4, 5 and 6 buffers were added to the support. The resulting suspension was incubated at 28°C–30°C under magnetic stirring (200 rpm) during 24 to 48 hours. Samples of 0.1 mL of supernatant were taken periodically, and the protein concentration in the supernatant was monitored at 280 nm until stabilization. Eupergit particles were recovered by centrifugation and washed with 5 mL acetate buffer pH 4.5, 0.1 M, and the supernatant was discarded. The support with enzyme was stored at 4°C until use. Aliquots of washing buffers and of supernatants were collected to establish immobilization efficiency.

#### 2.3.5. *β*-Glucosidase Immobilization in Amberlite IRC 50 and Amberlite IRC 86

Immobilization was basically performed according to Obón et al. [31]. Briefly, 1.0 g of Amberlite was added to test tubes, and Amberlite particles were washed with 5 mL of distilled water. The resulting suspension was centrifuged at 4000 rpm during 10 minutes at room temperature. The supernatant was discarded, the precipitate was washed with 5 mL of acetate buffer 0.1 M, pH 4.5, and the supernatant again discarded. 2.5 mL of a polyethyleneimine solution (100 g/L) were then added to the precipitate, and the suspension was incubated at room temperature under stirring during 2 hours. The mixture was centrifuged, the precipitate was washed with distilled water, and centrifuged again, and the supernatant was discarded.

5 mL of a glutaraldehyde solution 10% (v/v) were added to the tubes containing the support, and the mixture was incubated during 16 hours under stirring at room temperature. The tubes were centrifuged at 4000 rpm during 10 minutes, the supernatant was discarded, and the precipitate was washed with 5 mL of acetate buffer 0.1 M, pH 4.5. The suspension was filtered through qualitative filter paper, and 1.0 mL of enzymatic solution in acetate buffer pH 4.5; 0.1 M (0,1 g/L) was added to the activated Amberlite. The suspension was incubated at 28°C–30°C under stirring (200 rpm) during two hours then was centrifuged, and the precipitate was washed twice with acetate buffer pH 4.5; 0.1 M. The support with enzyme was maintained at 4°C until use. Aliquots of washing buffers and of supernatants were collected to establish encapsulation efficiency.

#### 2.3.6. *β*-Glucosidase Immobilization in Gelatin

Immobilization was basically performed according to Assis and co-workers [32]. To tubes containing 1 g of gelatin (Merck) 10 mL of acetate buffer pH 4.5, 0.1 M were added, and the resulting mixture was heated to dissolve the gelatin. Then, 2 mL of gelatin solution was transferred to another tube, and 200 *μ*L of enzyme solution in acetate buffer pH 4.5; 0.1 M (0,1 g/L) were added, and the whole was thoroughly mixed under magnetic stirring. The mixture was transferred to a Petri dish and stored at 5°C during 1 hour to allow for solidification. 4 mL of a glutaraldehyde solution (10%, v/v) were added above the gelatin layer for promoting cross-linking, and the whole was maintained at 5°C during 1 hour. The supernatant was then discarded, and the gelatin was cut with a scalpel in cubic shaped particles of similar sizes (roughly 2 mm), which were maintained in acetate buffer pH 4.5; 0.1 M at 5°C, until use. Aliquots of washing buffers and of supernatants were collected to establish encapsulation efficiency.

### 2.4. Determination of *β*-Glucosidase Activity

The determination of *β*-glucosidase activity of both free and immobilized biocatalyst was performed according to Matsuura and co-workers [33]. The spectrophotometric method is based on the determination of p-nitrophenol released from the enzymatic hydrolysis of p-NPG in acetate buffer-based reaction medium. Reaction mixtures contained 0.3 mL 5 mM p-NPG in sodium acetate buffer 0.05 M pH 5.0 and an appropriate amount of free or immobilized *β*-glucosidase in 0.3 mL sodium acetate buffer. Reaction mixtures were incubated at 50°C for 15 min with 400 rpm magnetic stirring, followed by the addition of 0.3 mL 0.5 M Na_2_CO_3_ solution, pH 12, to stop the reaction. These conditions were established after preliminary confirmation that the initial rate of product formation was linear, therefore allowing for a simple calculation of the initial reaction rate based on single datum point, according to a methodology suggested by Doig and co-workers [34]. Hydrolysis was determined by monitoring the release of p-nitrophenyl at 410 nm with reference to a standard curve prepared using p-nitrophenol. Activity is expressed in international units (IUs), where 1 IU corresponds to the release of 1 *μ*mol p-nitrophenol per min. All runs were performed in triplicate, at least.

### 2.5. Immobilization Yield

The immobilization yield was calculated through *β*-glucosidase activity balance. Activity was determined according to 2.4.

### 2.6. PH and Temperature Profile

The activities of free and immobilized enzyme were compared. To observe the effect of the temperature, the tubes containing substrate were incubated under temperatures ranging from 40°C to 80°C. The effect of the pH in the enzymatic activity was determined by incubating the bioconversion medium in acetate buffer solutions (pH 4.0 to 6.0). The conditions of enzymatic assays were performed according to 2.4.

### 2.7. Kinetic Parameters

The effect of substrate concentration in the immobilized and free *β*-glucosidase activity was tested in different concentrations of p-NPG. The assays were performed under optimal pH and temperature. The *K_M_* (Michaelis constant) and *V*_max_ (maximum reaction rate) values were determined through Lineweaver-Burk or Hanes-Woolf linearization and through nonlinear method using the Solver Excel tool.

### 2.8. Stability Evaluation

The thermal and pH stabilities of free and immobilized enzymes were examined by measuring the activity of enzyme, determined as described in 2.4., after incubation of enzyme preparations in buffer solutions for 1 to 3 hours, at different temperatures (40°C–70°C) and pH values (4.0-5.0).

### 2.9. Repeated Batch Hydrolysis

Consecutive batch runs were performed under the conditions described in 2.4, at 50°C, pH 4.5, and an initial concentration of p-NPG of 5 mM. After each cycle, the immobilized biocatalyst was harvested, thoroughly washed with acetate buffer, and used for the next run.

## 3. Results and Discussion

### 3.1. Immobilization Yield

**Figure 1:**
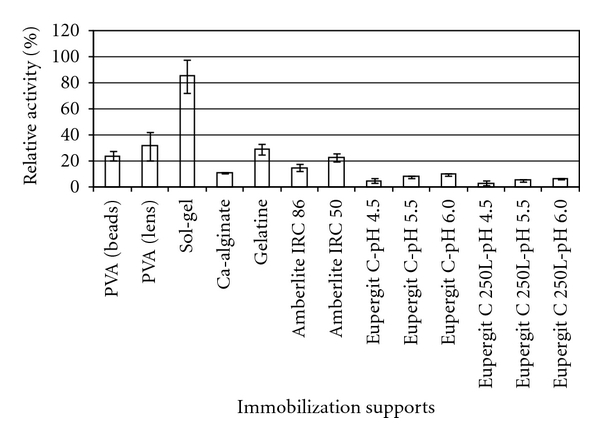
Immobilization efficiency of *β*-glucosidase for the supports screened.

Within the supports screened, the best results regarding immobilization yield were obtained for sol-gel encapsulation roughly in excess of 80% (Figure 1).

Immobilization in lenticular shaped Lentikats particles proved marginally more successful than in bead-like particles, the former exceeding 30%, whereas the later was a little over 20%. Encapsulation yield in gelatin was slightly lower than in lenticular shaped particles. Calcium alginate proved the least efficient support among gel-type supports. This could be ascribed to leakage of enzyme from the gel, which has been shown to be particularly relevant for most hydrogels, namely, for calcium alginate supports [7, 35]. The composition of the sol-gel used is likely to present a relatively low pore size, particularly when compared to hydrogels although some controversy exists on this matter on the effect of the nature of the sol-gel precursors on the pore size of the sol-gel particles [36–39]. Immobilization by binding to Amberlite or to Eupergit led to poorer results than those observed for entrapment methods safe for alginate. Glutaraldehyde used for immobilization in Amberlite could have a deleterious effect on the activity of the enzyme, hence the relatively low yields observed [40, 41]. When Eupergit is considered, an increase in efficiency can nevertheless be observed with the increase of the pH of the incubation media used for immobilization. Still, the increase in immobilization yield was nevertheless quite mild with pH, and even at pH 6.0, yields were still quite minute as compared with the other methods screened. Since Eupergit is known to bind to proteins through the oxirane groups of the support, that react with the amino groups of the protein molecules at neutral and alkaline pH, or with the sulfhydryl groups and carboxyl groups in the acidic, neutral, and alkaline pH range [9], it can be suggested that the former binding method should be favored, and eventually, immobilization in increasingly alkaline media would favor immobilization. All matters considered, and since some fungal glucosidases present the best operational stability at pH 4 to 6 [42], the optimal pH for activity is within 3 to 7 [43], no further research efforts were made, at the present stage, on the matter of immobilization in Eupergit. In a previous published works with a commercial *β*-glucosidase, Novozyme 188, immobilized in Eupergit C, the reported immobilization efficiency was 12%, roughly in accordance with the present work although with the use of additives this could be increased to 30% [44]. Several other supports were also screened for immobilization of Novozym 188, namely, activated charcoal, nylon, chitosan, bentonite, kaolin, silica gel, and titanium dioxide, but the authors only considered promising for further work immobilization on silica gel and on kaolin, where immobilization efficiencies of 35% and 95% were reported [45]. Screening of supports for the immobilization of a *β*-glucosidase enzyme preparation, Cytolase PCL 5 from Genencor, was also reported. Cellulose PEI, alpha- and gamma-alumina, and chitosan, occasionally functionalized with 3-aminopropyl-trimethyoxysilan (APTS) supports, were tested, with immobilization yields within 1.3% and 18% [46]. Immobilization of *β*-glucosidase from *Pyrococcus furiosus* in gelatine gel by cross-linking with transglutaminase allowed immobilization yields within 25 to 39 although when *β*-glucosidase from almonds was immobilized, the yield was only of 5% [47]. As compared with this previous information, the results gathered in the present work, namely, when sol-gel immobilization is concerned, lens-shaped particles, Lentikats, seemed also promising, and both were, therefore, selected for characterization.

### 3.2. Temperature and PH Profiles

**Figure 2:**
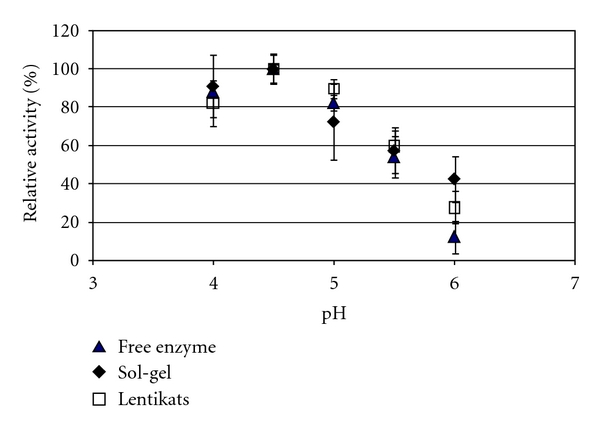
Effect of pH in the activity of free (triangle), sol-gel (diamonds), and Lentikats (squares) immobilized *β*-glucosidase. Bioconversion runs were performed at 50°C.

**Figure 3:**
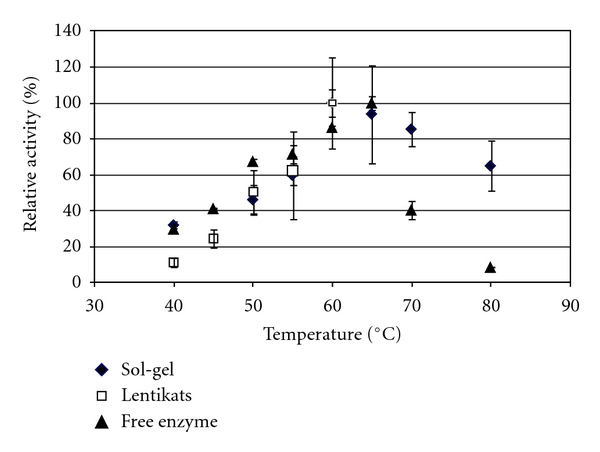
Effect of temperature in the activity of free (triangles), sol-gel (diamonds), and Lentikats (squares) immobilized *β*-glucosidase. Bioconversion runs were performed at pH 4.5.

The effect of immobilization in the initial reaction rate of p-NPG hydrolysis was evaluated within a given range of pH (Figure 2) and temperature (Figure 3). The immobilization in either sol-gel or Lentikats hardly altered the enzymatic pH-activity profile, as compared to the free form, with the pH optimum remaining unaltered at 4.5.

Only the activity decay of the free enzyme was slightly more pronounced at higher pH values, a feature also observed by Nagatomo and co-workers [47]. This can be tentatively ascribed to the protective role of the microenvironment surrounding the biocatalyst.

Roughly similar patterns, where optimum pH profile is not significantly altered with immobilization, were reported previously [44, 47] Martino and co-workers, on the other hand, observed a shift of the pH optimum from 5.0 to 4.0 as result of immobilization in chitosan [46]. Chang and Juang also reported a shift towards a more acidic environment as a result of immobilization in chitosan-clay composites [40].

The enzymatic temperature-activity profile displayed significant differences for the three forms of the biocatalyst (Figure 3). 

Lentikats biocatalyst proved effective up to 55°C, with no enzyme leakage observed, but above this temperature, melting of the support was observed. This later behavior was also reported [19] and prevented further evaluation of this support at higher temperatures, namely, up to 65°C which could be considered the optimum temperature for the activity of the free enzyme and sol-gel formulation. Above this temperature, there is a sharp decay of activity of the free enzyme, unlike what is observed for the sol-gel entrapped enzyme, which still retains about 65% of the initial activity at 80°C. A similar pattern was observed by Nagatomo and co-workers [47]. Chang and Juang also reported a higher tolerance range to heat of the clay composite immobilized *β*-glucosidase when compared to the free enzyme. The optimal temperatures of free and immobilized enzymes were within 55°C and 60°C. [40]. Martino and co-workers [46] and Synowiecki and Wołosowska [48] also reported an increased tolerance towards heat as a result of immobilization albeit without shifts in the optimal temperature.

### 3.3. Determination of Kinetic Parameters

The *K_M_* value of the immobilized enzyme was increased around 2- and 4.8- fold to sol-gel and Lentikats, respectively, when compared with the *K_M_* of free enzyme (Table 1), suggesting that the immobilization decreased the apparent affinity to the substrate, most likely as a result of diffusion limitations.

A roughly 4-fold increase in the *K_M_* was also reported as a result of *β*-glucosidase on chitosan [46]. Increased *K_M_* as a result of immobilization was also reported for immobilization of *β*-glucosidase on Eupergit C albeit cellobiose was used as substrate [44].

### 3.4. Thermal and pH Stability

**Figure 4:**
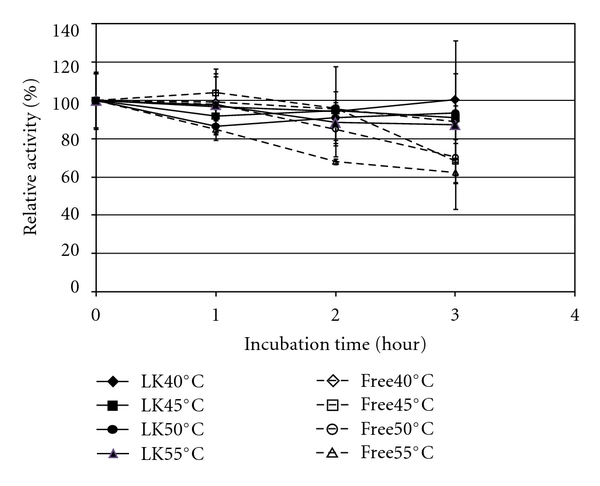
Thermal stability of free (free, open symbols) and Lentikats immobilized (LK, closed symbols) *β*-glucosidase. Runs were performed at pH 4.5.

Thermal stability was improved by immobilization in Lentikats, since after 3 hours of incubation, no significant loss of activity was observed irrespectively of the temperature used (Figure 4). An activity decrease for the free form of the enzyme was observed for temperatures in excess of 40°C, particularly noticeable when incubation was performed at 55°C, where a roughly 40% activity decay was observed after a 3-hour incubation period. Furthermore, this result was marginally lower than when incubation was performed under 45°C and 50°C.

**Figure 5:**
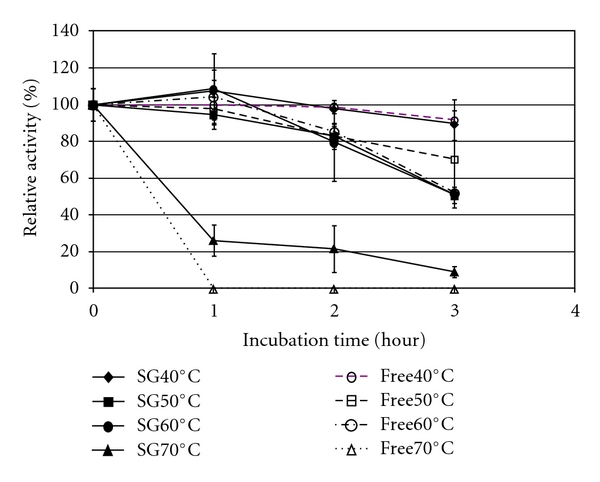
Thermal stability of free (free, open symbols) and sol-gel immobilized (SG, closed symbols) *β*-glucosidase. Runs were performed at pH 4.5.

The thermal stability of the sol-gel immobilized enzyme was evaluated in a broader range of temperature (Figure 5), given the higher physical stability of the sol-gel material to temperature, as compared to Lentikats. However, the stabilizing effect of the support was only noticeable for the highest temperature tested. Thus, under incubation at 70°C, the free enzyme was rapidly denatured, while the immobilized enzyme still retained 20% of the initial activity after 2 hours.

The immobilization support can have a protecting effect which may result of the changes in the conformational flexibility of the enzyme as an outcome of immobilization. The immobilization step increases enzyme rigidity, commonly reflected by an increase in stability towards thermal denaturation [49]. 

**Figure 6:**
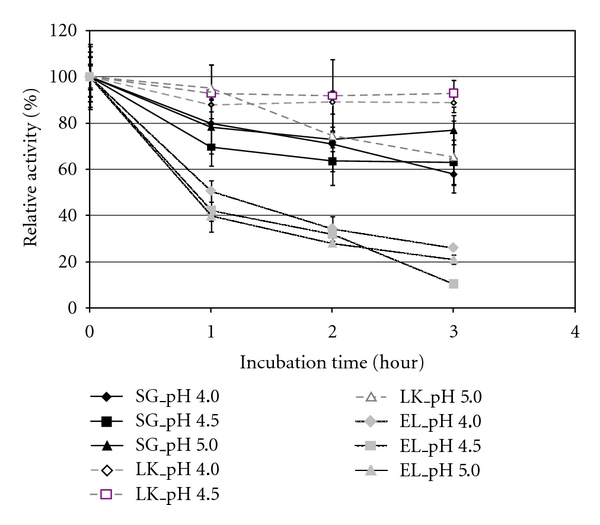
pH stability of free, sol-gel, and Lentikats immobilized *β*-glucosidase. Runs were performed at 55°C.

The pH stability was strongly improved following immobilization with both methods (Figure 6).

The enzyme entrapped in Lentikats was more stable in pH 4.0 and 4.5, with no significant activity decay, retaining around 90% of relative activity after 3 hours. However in pH 5.0 the relative activity was 65%. 

The behavior of the enzyme entrapped by the sol-gel method was very similar for pH 4.0 and 4.5, retaining around 60% of relative activity after 3 hours. Incubation at pH 5.0 favored stability, because the final relative activity was about 75% of the initial value.

### 3.5. Operational Stability

**Figure 7:**
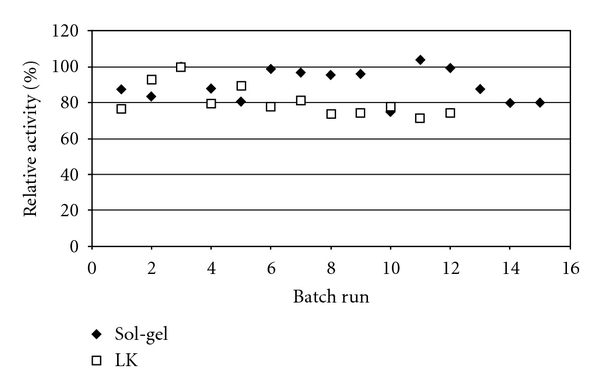
Effect of the repeated use of immobilized *β*-glucosidase in sol-gel (diamonds) and Lentikats (squares) on relative activity. Batch runs were performed at 50°C and pH 5.0. Standard deviation did not exceed 10%.

The selected supports were reused in consecutive 15 minutes batch runs using p-NPG synthetic substrate as reaction medium, and the activity of the immobilized enzyme established throughout the different runs (Figure 7). The possibility of the reuse of immobilized enzyme preparations is important, because this is a key feature for the economic viability of bioprocesses anchored in immobilized enzyme systems [50].

The different immobilized enzyme formulations tested were stable for more than 10 batch runs, suggesting the potential for application in systems with industrial relevance (namely, cellobiose hydrolysis). Chang and Juang [40] also reported on the possibility of the reuse of chitosan immobilized *β*-glucosidase, but these authors performed the runs in a clearly suboptimal temperature, 25°C, whereas the optimal temperature for activity was within 55°C to 60°C.

## 4. Conclusions

As a result of screening of different commercially available supports and methodologies for the immobilization of *β*-glucosidase, entrapment in sol-gel beads emerged as the most promising approach although Lentikats lenses also displayed potential for prospective applications. Neither method led to cant changes in the pH/activity profile, but the activity decay of the free enzyme was slightly more pronounced for pH 6. Entrapment in sol-gel did not result in significant changes in the optimal temperature, but immobilization resulted in a higher tolerance towards higher temperatures. Lentikats could only be used in suboptimal temperatures, since lenses were not physically stable beyond 55°C. In both methods, mass transfer limitations were observed, more noticeably in Lentikats, possibly given the larger size of the particles. Both methods enhanced the thermal stability of *β*-glucosidase, and both supports were used in consecutive batch runs without activity decay.

These results suggest that these methods have potential for the use of immobilized *β*-glucosidase in industrially relevant processes, namely, hydrolysis of cellobiose. Achieving such goal will require further significant work in order to evaluate the feasibility of these approaches under process conditions using said substrate.

## Figures and Tables

**Table 1:** Kinetic parameters for free and immobilized *β*-glucosidase. Standard deviation did not exceed 10%.

Biocatalyst	Lineweaver-Burk		Hanes-Woolf		Solver	
	*K_M_* (mM)	*V*_max_ (mM L^−1^ min^−1^)	*K_M_* (mM)	*V*_max_ (mM L^−1^ min^−1^)	*K_M_* (mM)	*V*_max_ (mM L^−1^ min^−1^)
Free enzyme	1.4	0.02	1.54	0.02	1.6	0.02
Sol-gel	5.0	0.44	8.06	0.20	7.33	0.63
Lentikats	12.0	0.27	6.14	0.58	4.16	0.17
